# Exploring the Application of Machine Learning for Daily‐Life Near‐Fall Detection in Older Adults

**DOI:** 10.1111/ggi.70862

**Published:** 2026-10-15

**Authors:** Nina Marie Schmidt, Tania Zieschang, Lea Feld, Laura Himmelmann, Elisa‐Marie Speckmann, Tim Stuckenschneider

**Affiliations:** ^1^ Geriatric Medicine, Department for Health Services Research, School of Medicine and Health Sciences Carl von Ossietzky University Oldenburg Germany; ^2^ Department for Health Services Research, Assistance Systems and Medical Device Technology Carl von Ossietzky University Oldenburg Germany; ^3^ Department of Medical Physics and Acoustics, Medical Physics and Cluster of Excellence Hearing4all Carl von Ossietzky University Oldenburg Germany

**Keywords:** gait parameters, gait perturbations, sensors, slips and trips

## Abstract

**Background:**

Falls are a health concern among older adults, and near‐falls may serve as an early indicator of future falls, functional decline, and the need for targeted preventive interventions. While previous research has successfully demonstrated near‐fall detection using machine learning in controlled laboratory environments, it remains crucial to evaluate the application of such approaches in daily‐life settings.

**Aim:**

This study examines the application of a pre‐trained machine learning algorithm to detect gait perturbations in daily‐life and to explore how these classified gait perturbations are associated with participant characteristics.

**Methods:**

Data from the SeFallED study was used, which followed up on individuals aged ≥ 60 years who presented to the emergency department following a fall. Daily stepping time was recorded using a thigh‐worn accelerometer over 7 days, segmented into windows, and analyzed using a laboratory‐based pre‐trained one‐dimensional convolutional neural network to detect near‐falls.

**Results:**

A total of 202 participants (median age 75 years) were included in the analysis. On average, 58% of total stepping time windows were classified as gait perturbation windows. Higher age, lower cognitive and functional performance, greater concerns about falling, and higher levels of frailty were associated with a greater proportion of classified gait perturbations.

**Conclusion:**

The laboratory‐trained algorithm classified a high number of windows as gait perturbations, likely reflecting gait irregularities including misclassifications rather than exclusively near‐falls. Although these classifications still meaningfully reflect participant characteristics, further training and validation are required to enhance the algorithm's ability to accurately distinguish and objectively assess gait perturbations and near‐falls in daily‐life.

## Graphical abstract

A laboratory‐trained machine learning algorithm analyzed daily‐life thigh sensor data in older adults after a fall. It classified gait perturbation windows as near‐fall proxies, which were associated with clinical risk factors. However, classified windows likely reflect gait irregularities rather than near‐falls, highlighting the need for daily‐life validation and model refinement.

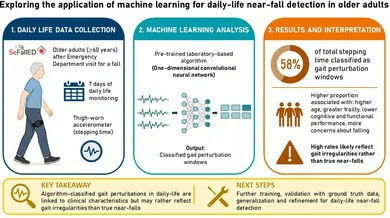

## 1 Introduction

With a global annual prevalence of approximately 26.5%, falls are a health concern among older adults [1], as they are often associated with adverse outcomes [2, 3, 4]. Exercise‐based programs have been shown to be effective in falls prevention [5]. However, these programs often fail to reach those at highest risk, possibly reflecting a lack of early identification. Therefore, early detection of individuals at risk for falls is crucial to enable their integration into targeted preventative interventions [6].

Among the various risk factors for falls, near‐falls play an important role. They are defined as gait perturbations involving tripping, slipping, or other losses of balance, in which individuals successfully recover, thereby preventing an actual fall [7]. These events are prevalent among older adults [8, 9] and are strongly associated with an increased risk of falls. A study with 60 older adults found that approximately 40% experienced near‐falls within 3 months, and that these events significantly increased the risk of future falls, highlighting their prognostic value for fall risk assessments [10]. Similarly, a study of 266 older adults showed a significant association between self‐reported near‐falls and falls [7]. Notably, near‐falls occurred three times more frequently than actual falls, suggesting that near‐falls may precede falls and could serve as an early marker for identifying individuals at increased risk. However, as near‐falls were assessed through self‐report, the data are subject to recall and reporting bias. Developing objective and automated methods for near‐fall detection could enable the investigation of near‐falls as an early indicator for fall risk.

Previous research has attempted to detect near‐falls using machine learning, which showed great promise in controlled, laboratory‐based environments. Two studies [11, 12] investigated algorithms in younger adults using inertial measurement units (IMUs) and laboratory‐induced perturbations, while a third study [13] included both younger and older adults. Another study by Wang et al. [14] focused on healthy older adults, using accelerometer data to analyze laboratory‐induced slips. All studies reported successful and precise detection of near‐falls. Focusing on a more heterogeneous population, Hellmers et al. [15] detected near‐falls in 87 older adults with a history of falls that were exposed to treadmill gait perturbations. The best results were achieved with an upper thigh‐worn accelerometer, where the one‐dimensional convolutional neural network with data augmentation (1D‐CNN + DA) yielded a precision of 0.838 ± 0.081, recall of 0.867 ± 0.042, and an F1‐score of 0.851 ± 0.054. In this context, transferring lab‐trained models to daily‐life introduces a domain shift, as movement patterns differ substantially between settings [16]. This may affect classification performance in daily‐life applications.

Although laboratory settings are limited by restricted external validity, particularly in reproducing unexpected near‐fall events, this highlights the importance of monitoring near‐falls in daily‐life, yet only a few studies have addressed this objectively. Iluz et al. [17] detected near‐falls in 40 individuals with Parkinson's disease using a single sensor, while another study detected 18 trip events in five older adults using multiple IMUs [18].

While previous studies provide insights into near‐fall detection outside the laboratory, their limitations, such as short recording durations, small sample sizes, and restricted types of near‐falls, highlight the challenge of translating lab‐based algorithms to daily‐life environments. For daily‐life applications, algorithms should plausibly reflect established risk factors such as advanced age, cognitive impairment, concerns about falling (CaF), and frailty. These factors have been shown to increase both the likelihood of falls and near‐falls [19, 20]. This study addresses the existing gap in near‐fall detection by exploring the transferability of a lab‐based pre‐trained algorithm to daily‐life conditions, as an initial step toward clinical translation. Since true near‐fall events are difficult to verify in daily‐life environments, associations between algorithm‐classified near‐fall events (i.e., classified gait perturbation windows) and participant characteristics will be examined. Moreover, comparisons across different age groups, cognitive performance levels, CaF, and degrees of physical frailty will be conducted to further explore the potential of wearable sensors for assessing fall risk. It is hypothesized that the number of near‐falls are positively linked with higher age, lower cognitive performance, higher CaF, and a higher level of frailty.

## 2 Methods

### 2.1 Participants

Participants were recruited from the SeFallED study [21], which investigated the long‐term trajectories in activities of daily living of older adults who presented to the emergency department (ED) following a fall. Ethical approval was obtained from the Medical Ethics Committee of the University of Oldenburg (approval number 2021–120). Participants were eligible for inclusion if they were aged 60 years or older, were admitted to the ED due to a fall, and were discharged within 72 h. The recruitment process, inclusion, and exclusion criteria were previously described in the published study protocol [21]. For this sub‐study, participants were excluded if they did not consent to wear an accelerometer or if they accumulated fewer than five valid wear days (non‐wear periods of up to 4 h per 24‐h recording were considered valid according to the manufacturer's default settings), which is in line with previous research [22].

### 2.2 Study Overview

Participants were recruited in the ED following their fall [23]. After obtaining informed consent, they were visited at home within 4 weeks of the initial fall for a comprehensive geriatric assessment. The assessments included standardized questionnaires and physical performance tests, followed by the placement of an accelerometer (activPAL, PAL Technologies Ltd., Glasgow, UK; sampling frequency 40 Hz) on the right upper thigh using a waterproof adhesive dressing. This sensor was worn continuously for seven consecutive days to monitor physical activity. The sensor was only removed for swimming.

### 2.3 Data Processing

Accelerometer data from the activPAL were processed using PALanalysis (PAL Software Suite, PAL Technologies Ltd., Glasgow, UK), categorizing the recorded movements into physical activity types. Only periods classified as stepping time with a minimum duration of 2 s were selected for further analysis and segmented into 2‐s windows using a sliding window approach. For further analysis, the pre‐trained 1D‐CNN + DA from Hellmers et al. [15] was applied. The original probability threshold of 0.5 was retained to classify each window as either a regular gait pattern (predicted value < 0.5) or a gait perturbation (predicted value ≥ 0.5). Detailed information on the architecture, training procedure, and performance of the algorithm can be found in the publication by Hellmers et al. [15].

### 2.4 Assessment Dimensions

Participants' characteristics were recorded, including demographic and clinical information such as sex, age, fall history, number of diseases, and years of education. Additionally, cognitive and mental assessment, as well as functional and activity‐related assessment, were assessed using standardized and validated instruments. Classified gait perturbation windows derived from the algorithm were expressed as the percentage of classified gait perturbation windows within all applied windows. Details on the operationalization, range, and interpretation of all assessment dimensions included in this sub‐study are provided in Table 1.

**TABLE 1:** Assessment dimensions and their operationalization.

Dimensions	Measure	Operationalization (range and interpretation)
Cognitive and mental assessments	Montreal Cognitive Assessment (MoCA) [24]	0–30 points; higher scores indicate greater cognitive performance; scores are categorized as no cognitive impairment (> 24 points) and probable cognitive impairment (≤ 24 points) [25]
	Trail Making Test A and B (TMT A + B) [26]	Time in seconds; lower values indicate better processing speed, visual attention, cognitive flexibility, and executive functioning
	Short Falls Efficacy Scale‐International (FES‐I) [27]	7–28 points; higher scores indicate greater concerns about falling; scores are categorized as low concerns (7–8 points), moderate concerns (9–13 points), and high concerns (14–28 points)
Functional and activity‐related assessment	Barthel Index [28]	0–100 points; higher scores indicate greater independence in activities of daily living
	Longitudinal Urban Cohort Ageing Study‐Function Index (LUCAS‐FI) [29]	1–4 points; scores are categorized as robust (1 point), post‐robust (2 points), pre‐frail (3 points), and frail (4 points); higher scores indicate increasing frailty
	Short Physical Performance Battery (SPPB) [30]	0–12 points; higher scores indicate better lower extremity function
	Grip strength [31]	Measured in kilograms
	Physical Activity Questionnaire‐50 (PAQ‐50) [32]	Metabolic equivalent (MET) per week; higher scores indicate grater self‐reported physical activity levels
	Accelerometer (activPAL, PAL Technologies Ltd., Glasgow, UK)	Worn for 7 days on the upper thigh recorded at 40 Hz and processed using PALanalysis (PAL Software Suite, PAL Technologies Ltd., Glasgow, UK)
	Step count Stepping time Sedentary time	Average number of steps per day Average stepping minutes per day Average sedentary minutes per day

### 2.5 Statistical Methods

All measures are presented as medians with interquartile ranges (IQRs). Associations between the proportion of classified gait perturbation windows and participant characteristics were examined using Spearman's rank correlation coefficients. Effect sizes were interpreted according to Cohen, with *r* values of 0.1, 0.3, and 0.5 representing small, medium, and large effects, respectively [33]. Group differences were analyzed using Mann–Whitney *U* test or Kruskal–Wallis test, as data were not normally distributed. When a significant effect was observed, Bonferroni‐corrected pairwise Mann–Whitney *U* post hoc tests were conducted to identify between‐group differences. Comparisons were performed across age groups (≤ 74, 75–84, and ≥ 85 years), cognitive performance groups (MoCA; no cognitive impairment, > 24 points; probable cognitive impairment, ≤ 24 points), levels of concerns about falling (FES‐I; low concerns, 7–8 points; moderate concerns, 9–13 points; and high concerns, 14–28 points), and frailty status (LUCAS‐FI; robust; post‐robust; pre‐frail; and frail). Statistical significance was set at *p* ≤ 0.05. All analyses were conducted using IBM SPSS Statistics, version 30 (IBM Corp., Armonk, NY, USA).

## 3 Results

### 3.1 Participant Characteristics

Of the 335 participants of the SeFallED study a total of 202 participants aged 60–93 years (median = 75 years, IQR = 66.5–81.5) were included in this sub‐study. The median total stepping time was 624.3 min (IQR = 399.4–860.7), yielding a median of 17 196 input windows (IQR = 10 710–23 980) per participant. In median, 58% (IQR = 48.2–71.2, min = 11.8, max = 99.9) of the windows were classified as gait perturbations. Details on participants' demographic, clinical, cognitive and mental, as well as functional and activity‐related characteristics are provided in Table 2.

**TABLE 2:** Participants demographics and clinical characteristics, cognitive and mental as well as functional and activity‐related assessments.

	*n*	%	Median (IQR)	Min	Max
Demographic and clinical characteristics					
Total number of participants	202				
Sex (female/male)	129/73	63.9/36.1			
Age (years)	202		75 (66.5–81.5)	60	93
≤ 74	86	42.6	67 (63–72)	60	74
75–84	77	38.1	80 (77–82)	75	84
≥ 85	39	19.3	87 (85–89)	85	93
Fall history (number of falls within 12 months)	202		1 (1–1)	0	19
Number of diseases	202		2 (1–4)	0	11
Education (school years)	202		10 (9–12)	5	16
Cognitive and mental assessments					
MoCA	194		25 (22–27)	11	30
No cognitive impairment	108		26 (25–28)	24	30
Cognitive impairment	86		21 (19–23)	11	23
TMT A	190		39.1 (31.0–54.1)	31.00	251.63
TMT B	183		107.0 (80.7–168.5)	38.71	301
FES‐I	198		8 (7–10)	7	28
Low concerns	97	49.0	7 (7–8)	7	8
Moderate concerns	73	36.9	10 (9–12)	9	13
High concerns	28	14.1	16 (14–20.8)	14	28
Functional and activity‐related assessments					
Barthel Index	198		100 (100–100)	30	100
LUCAS‐FI	197		2 (1–4)	1	4
Robust	95	48.2			
Post‐robust	19	9.6			
Pre‐frail	27	13.7			
Frail	56	28.4			
SPPB	177		10 (8–11)	1	12
Grip strength	181		26.3 (19.2–35.5)	1	60
PAQ‐50	192		94.6 (51.3–128.3)	0	343.4
Step count	202		5621 (3516–8053)	106	18 394
Stepping time	201		79.4 (52.7–111.7)	2.65	218.73
Sedentary time	202		646.2 (555.4–727.0)	326.94	1203.02

Abbreviations: FES‐I, Falls Efficacy Scale‐International; IQR, Interquartile Range; LUCAS‐FI, Longitudinal Urban Cohort Ageing Study‐Function Index; MoCA, Montreal Cognitive Assessment; PAQ‐50, Physical Activity Questionnaire‐50; SPPB, Short Physical Performance Battery; TMT, Trail Making Test.

### 3.2 Correlation Analysis

The results of the correlation analysis are presented in Table 3. Medium correlations were observed for age and education, indicating that a higher number of gait perturbation windows was associated with greater age and education. Furthermore, small positive correlations with cognitive function and medium positive correlations with attention and executive function were revealed. Again, better cognitive performance was associated with fewer gait perturbation windows. Similarly, higher CaF was correlated with a higher proportion of gait perturbation windows. Small to medium negative correlations were observed for Barthel Index, SPPB, grip strength, PAQ‐50, step count, and daily stepping time. These correlations indicate that lower activity levels and poorer functional performance were associated with higher gait perturbation windows. Small positive correlations were found for LUCAS‐FI and sedentary time.

**TABLE 3:** Spearman correlations between participant characteristics and classified gait perturbation windows.

	*n*	*r*	*p*
Demographic and clinical characteristics			
Age (years)	202	0.393	< 0.001**
Fall history (number of falls within 12 months)	202	0.083	0.240
Number of diseases	202	0.056	0.427
Education (school years)	202	−0.256	< 0.001**
Cognitive and mental assessments			
MoCA	194	−0.204	0.004**
TMT A	190	0.319	< 0.001**
TMT B	183	0.265	< 0.001**
FES‐I	198	0.242	< 0.001**
Functional and activity‐related assessments			
Barthel Index	198	−0.323	< 0.001**
LUCAS‐FI	197	0.213	0.003**
SPPB	177	−0.238	0.001**
Grip strength	181	−0.285	< 0.001**
PAQ‐50	192	−0.352	< 0.001**
Step count	202	−0.325	< 0.001**
Stepping time	201	−0.321	< 0.001**
Sedentary time	202	0.157	0.026*

*Note:* *r* values of 0.1, 0.3, and 0.5 representing small, medium, and large effects, respectively.

Abbreviations: FES‐I, Falls Efficacy Scale‐International; LUCAS‐FI, Longitudinal Urban Cohort Ageing Study‐Function Index; MoCA, Montreal Cognitive Assessment; PAQ‐50, Physical Activity Questionnaire‐50; SPPB, Short Physical Performance Battery; TMT, Trail Making Test.

* *p* > 0.05.

** *p* < 0.01.

### 3.3 Group Comparison

Detailed results of the group comparisons are presented in Figure 1 and Table S1. Significant differences were observed in individuals with probable cognitive impairment showing a higher proportion of perturbed windows compared to those without (*U* = 4156, *p =* 0.026). Across age groups, older participants exhibited a higher proportion of gait perturbation windows than younger participants (*H* = 29.861, *p* < 0.001). Participants with high CaF showed a greater proportion of gait perturbation windows compared to those with low concern (*p* = 0.012). Similarly, frail participants demonstrated a higher proportion of gait perturbation windows than robust individuals (*p* = 0.011).

**FIGURE 1:**
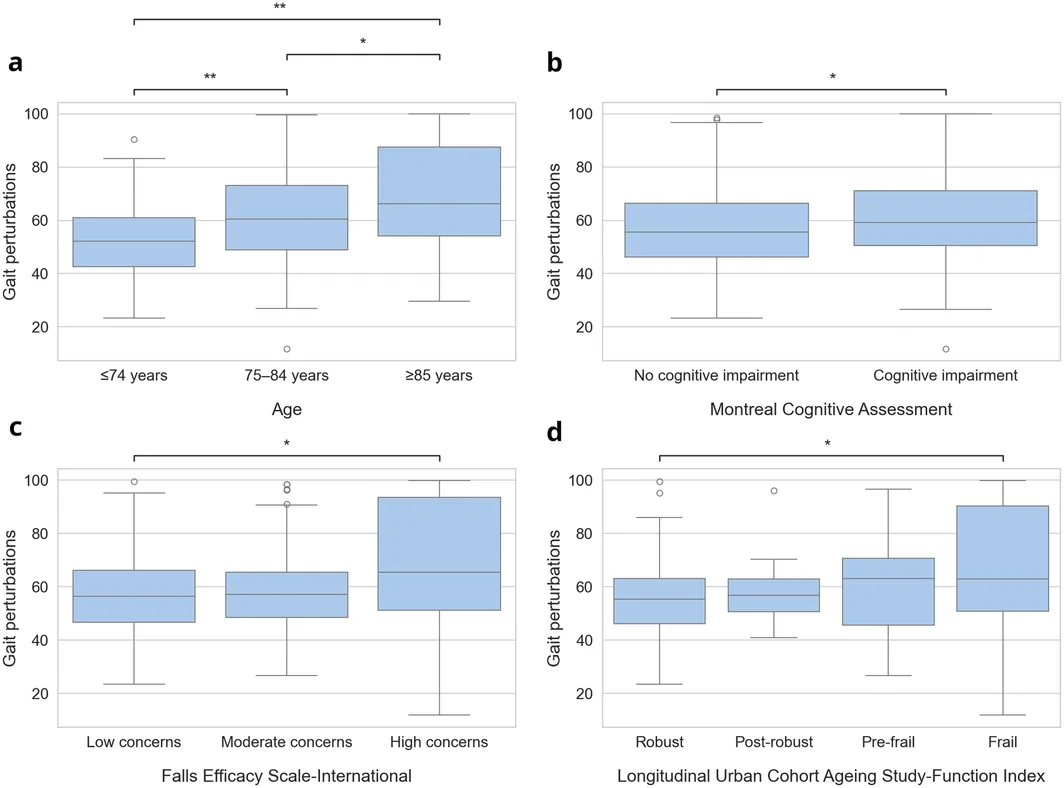
Group comparisons for gait perturbations across age, cognitive performance, concerns about falling and frailty. Group comparisons for classified gait perturbation windows across age (a), Montreal Cognitive Assessment (MoCA; b), Falls Efficacy Scale‐International (FES‐I; c), and Longitudinal Urban Cohort Ageing Study‐Function Index (LUCAS‐FI; d). Data are presented as boxplots showing the median, interquartile range, and outliers. The Mann–Whitney *U* test was used for MoCA. For age, FES‐I, and LUCAS‐FI, Kruskal–Wallis tests with Bonferroni‐corrected pairwise Mann–Whitney *U* post hoc tests were conducted. *p < 0.05, **p < 0.01.

## 4 Discussion

This study explored the application of a pre‐trained machine learning algorithm to daily‐life data. The purpose was to identify near‐falls across groups and to investigate associations with cognitive and mental assessments, as well as functional and activity‐related assessments. Correlation analyses showed that higher age, lower education, poorer cognitive and functional performance, reduced physical activity, and greater frailty were linked to a higher proportion of classified gait perturbation windows. Consistent with these findings, group comparisons revealed that a higher proportion of classified gait perturbation windows was observed in older participants, individuals with probable cognitive impairment, those with greater CaF, and frail participants.

### 4.1 Classified Gait Perturbation Windows

The algorithm classified a relatively high proportion of gait perturbation windows with a median of 58% per participant, exceeding numbers reported by previous studies. For example, one study that monitored individuals with Parkinson's disease with a shorter recording period of 3 days found 4.2%–24.3% of gait windows, representing suspected near‐falls [17]. Another study documented 71 near‐falls in 60 participants over 3 months using daily diaries [10], in a slightly older cohort, where only one third of participants reported a recent fall. In contrast, lower proportions of detected gait perturbation windows would be expected in our study population, due to the absence of neurological conditions and younger age. Another study involving five individuals with a fall history, comparable to the present study, identified between one and seven near‐falls per person over a longer 14‐day recording period [18]. These differences between previous studies and our results may reflect differences in study population, the algorithm's training data, and methodological approaches. The algorithm developed by Hellmers et al. [15] was trained on participants who underwent a protocol with fixed perturbation magnitudes. Consequently, fitter participants experiencing perturbations with small magnitudes likely required minimal compensatory responses, which were nonetheless included in the training data. This may have increased the algorithm's sensitivity to subtle deviations from stable gait, enabling detection of minor gait irregularities. Moreover, treadmill walking in controlled laboratory settings differs substantially from daily‐life walking, where small interruptions or changes in gait rhythm, such as responding to environmental distractions, are common but constrained on a treadmill. On a treadmill, individuals walk at constant speed with continuous gait cycles, leaving little opportunity for natural gait irregularities to occur. Similarly, Schmitt et al. [16], reported greater gait variability in outdoor walking compared to treadmill walking among older adults. Therefore, the treadmill‐trained algorithm may have classified naturally occurring daily‐life gait irregularities as gait perturbations, contributing to the high proportion of perturbed windows observed.

These findings highlight the need to refine, adapt, and ensure generalizability of algorithms for real world applications. Future training should incorporate a broader and more representative dataset, including a wider range of perturbation magnitudes to capture both subtle and pronounced compensatory responses. Integrating daily‐life walking data and overground perturbation scenarios, such as obstacle‐induced compensatory steps as demonstrated by Okubo et al. [34], could improve the algorithm's ability to differentiate between gait irregularities and perturbations indicative of increased fall risk. Additionally, future studies should aim to verify near‐fall events in daily‐life (e.g., video‐based methods or daily diaries), enabling evaluation and potential adjustment of the algorithm's probability threshold to improve classification robustness. Although the present approach is based on window‐based segmentation, verified events could enable more event‐based segmentation analyses in future work. Moreover, further research is required to determine whether actual near‐fall events are necessary to assess fall risk, or if subtle gait changes alone can serve as predictive markers of future falls.

### 4.2 Correlations

Correlation analyses revealed small to medium associations with functional and activity‐related assessments, largely in line with expectations. Prior literature has demonstrated strong links between overall physical functioning and gait characteristics [35]. Individuals with higher activity levels may exhibit better neuromuscular control, resulting in more efficient and less irregular gait patterns [36]. Other factors, such as increased sensory awareness, altered neuromuscular control, or residual pain after the fall, may also contribute to gait irregularities, highlighting the need for further research to elucidate the underlying physiological mechanisms, particularly for subtle gait changes. Assuming that the algorithm can detect subtle gait irregularities, long‐term monitoring combined with physiological assessments could provide valuable insights into the emergence of gait perturbations and their contribution to fall risk.

In contrast to findings from previous research [7, 10], fall history was not correlated with the classified gait perturbation windows. This finding may reflect characteristics of the study population, who were recruited from the ED following a fall. For some participants, this event may have been their first significant fall and severe enough to require medical attention, potentially causing temporal changes in gait, such as more cautious walking or pain‐related movement adaptations. Such fall‐related gait irregularities, as well as naturally occurring gait disturbances during daily‐life, may have resulted in misclassification and potentially attenuated the association with fall history. Additionally, the limited variability in the present sample, with most participants reporting a single fall, may reduce the ability to observe associations. Fall history was assessed retrospectively via self‐report and may therefore be prone to bias. Therefore, future studies should incorporate longitudinal fall data to examine how gait irregularities evolve over time and whether they more reliably predict prospective falls rather than past fall history.

### 4.3 Group Comparisons

Group comparisons further showed that higher age and greater frailty were associated with a higher proportion of classified gait perturbation windows. These findings are in line with previous research suggesting that aging and frailty are associated with gait changes, including slower, more cautious walking and increased gait variability. Such variability is widely recognized as an early marker of adverse outcomes, including an elevated risk of future falls [37, 38]. In addition, differences in cognitive performance and CaF were associated with gait perturbation windows. Cognitive impairment, particularly reduced executive functioning and attentional capacity, as well as high CaF, have consistently been linked to slower, less stable, and more irregular gait [39, 40]. As daily‐life walking requires continuous adaptation to environmental demands, limited cognitive capacity may lead to more frequent subtle gait irregularities. Similarly, individuals with elevated CaF may adopt a more cautious gait, likely resulting in increased gait irregularities that classify as perturbation windows. This effect may have been particularly pronounced in the present study, as participants were recruited shortly after presenting to the ED a fall. Future research should include participants both with and without a prior history of falls to more rigorously evaluate the algorithm. Although further refinement of the algorithm is required, as not all classified gait perturbations represent true near‐falls, this exploratory analysis demonstrates its potential to identify distinct risk profiles. Despite this, these preliminary findings are promising and support further investigation into the utility of wearable sensors, particularly for improving the training and validation of algorithms aimed at detecting near‐falls more accurately.

### 4.4 Strengths and Limitations

This study represents a novel approach to identify near‐falls in daily‐life using wearable sensors, but several methodological limitations should be acknowledged. As with most algorithms, the internal decision processes of the algorithm are not fully transparent, leaving uncertainty about which movement features are classified as gait perturbations. Moreover, applying an algorithm trained under controlled laboratory conditions to daily‐life environments introduces considerable variability. The high proportion of classified gait perturbation windows likely reflects misclassification of naturally occurring daily‐life gait irregularities or artifacts, highlighting the need for verification of near‐falls in daily‐life. It should also be noted that the study relied on a single algorithm, limiting the assessment of whether alternative algorithms could have produced different classification outcomes. The study population consisted of older adults presenting to the ED after a fall. While this may limit generalizability to the broader population, it represents a clinically relevant high‐risk group for future functional decline and recurrent falls. Despite these limitations, the automated, sensor‐based approach offers substantial advantages enabling analysis of large datasets without reliance on retrospective self‐report.

## 5 Conclusion

Although transferring the laboratory‐trained algorithm to daily‐life did not yet allow for the accurate detection of actual near‐falls, the algorithm classified gait perturbations that were meaningfully linked to participant characteristics. Further training, validation, and generalization of the algorithm are required to improve its performance in daily‐life settings. With further refinement, this approach has the potential to contribute to the development of objective methods for assessing near‐fall events in daily‐life. Such advancements may help identify individuals who experience these events more frequently and are at higher risk for future falls, facilitating earlier and more targeted falls prevention strategies in both clinical and community settings. Moreover, considering the rapidly evolving wearable technology industry, machine learning algorithms could facilitate longitudinal monitoring, potentially detecting early changes in gait.

## Author Contributions

T.Z., T.S., and N.M.S. were responsible for conceptualization. T.S. and N.M.S. carried out data curation and performed the formal analysis. T.Z. and T.S. were responsible for the investigation. N.M.S. and T.S. wrote the original draft of the manuscript and were responsible for visualization while T.Z., L.F., L.H., and E.‐M.S. reviewed and edited the manuscript. All authors have read and approved the final version of the manuscript and agreed with the order of presentation of the authors.

## Funding

Open Access funding enabled and organized by Projekt DEAL. This work was supported by the Federal Ministry of Education and Research (BMFTR, grant number: 01GY2021). The BMFTR did not contribute to designing the study or in writing the manuscript, nor has been involved in data collection, analysis, and interpretation.

## Ethics Statement

The authors state that their research was conducted ethically in accordance with the Declaration of Helsinki. This study was approved and reviewed by the Medical Ethics Committee of the University of Oldenburg, approval number 2021‐120.

## Consent

The participants or their legal guardian or family member provided their written informed consent to participate in the study.

## Conflicts of Interest

The authors declare no conflicts of interest.

## Supporting information


**Table S1:** Group comparisons for gait perturbations across age, cognitive performance, concerns about falling and frailty.

## Data Availability

The data that support the findings of this study are available on request from the corresponding author. The data are not publicly available due to privacy or ethical restrictions.
