# Supplementary material for: Exploring the Application of Machine Learning for Daily‐Life Near‐Fall Detection in Older Adults
Source: Geriatr Gerontol Int. 2026 Oct 15;26(10):e70862. doi: 10.1111/ggi.70862 (PMC13670406; doi:10.1111/ggi.70862)
Supplement: Supplementary file 1 [file GGI-26-0-s001.docx]

**Supplementary Material 1.**

Table 1. *Group comparisons for gait perturbations across age, cognitive performance, concerns about falling and frailty.*

|  | **n** | **Test Statistic** | **p-value** | **Group 1** | **Group 2** | **Group 3** | **Group 4** | **Post hoc** |
| --- | --- | --- | --- | --- | --- | --- | --- | --- |
| **MoCA** |  |  |  | **No cognitive impairment** | **Cognitive impairment** |  |  |  |
|  | 202 | 4156 | 0.026* | 56.1 (46.1 – 68.5) | 61.5 (50.7 – 79.4) |  | - | - |
| **Age** |  |  |  | **≤74 years** | **75 – 84 years** | **≥85 years** |  |  |
|  | 202 | 29.861 | <0.001** | 52.3 (42.1 – 62.1) | 61.2 (48.8 – 73.5) | 78.1 (55.8 – 90.9) | - | Gr. 1 - 2: 0.003**  Gr. 1 - 3: 0.000**  Gr. 2 - 3: 0.031* |
| **FES-I** |  |  |  | **Low concern** | **Moderate concern** | **High concern** |  |  |
|  | 202 | 8.252 | 0.016* | 56.7 (46.6 – 66.7) | 58.0 (48.5 – 70.6) | 71.9 (51.7 – 95.4) | - | Gr. 1 - 2: 0.978  Gr. 1 - 3: 0.012*  Gr. 2 - 3: 0.113 |
| **LUCAS-FI** |  |  |  | **Robust** | **Post-robust** | **Pre-frail** | **Frail** |  |
|  | 197 | 10.080 | 0.018* | 55.8 (46.2 – 63.2) | 56.7 (49.9 – 64.5) | 62.0 (45.3 – 71.0) | 63.1 (50.2 – 90.6) | Gr. 1 - 2: 1.000  Gr. 1 - 3: 1.000  Gr. 1 - 4: 0.011*  Gr. 2 - 3: 1.000  Gr. 2 - 4: 0.498  Gr. 3 - 4: 0.672 |

The Mann-Whitney U test was used for MoCA. For age, FES-I, and LUCAS-FI, Kruskal-Wallis tests with Bonferroni-corrected pairwise Mann-Whitney U post-hoc tests were conducted. MoCA, Montreal Cognitive Assessment; TMT, Trail Making Test; FES-I, Falls Efficacy Scale-International; LUCAS-FI, Longitudinal Urban Cohort Ageing Study-Function Index; SPPB, Short Physical Performance Battery; PAQ-50, Physical Activity Questionnaire-50. * p>.05, ** p<.01.
